# Identification of Polymorphisms in EAAT1 Glutamate Transporter Gene *SLC1A3* Associated with Reduced Migraine Risk

**DOI:** 10.3390/genes15060797

**Published:** 2024-06-18

**Authors:** Cassie L. Albury, Heidi G. Sutherland, Alexis W. Y. Lam, Ngan K. Tran, Rod A. Lea, Larisa M. Haupt, Lyn R. Griffiths

**Affiliations:** 1 Genomics Research Centre, Centre for Genomics and Personalised Health, School of Biomedical Sciences, Queensland University of Technology (QUT), 60 Musk Ave., Kelvin Grove, QLD 4059, Australia; calbury15@hotmail.com (C.L.A.); heidi.sutherland@qut.edu.au (H.G.S.); alexis.lam@qut.edu.au (A.W.Y.L.); k23.tran@qut.edu.au (N.K.T.); rodney.lea@qut.edu.au (R.A.L.); 2 Stem Cell and Neurogenesis Group, Genomics Research Centre, Centre for Genomics and Personalised Health, School of Biomedical Sciences, Queensland University of Technology (QUT), Kelvin Grove, QLD 4059, Australia; larisa.haupt@qut.edu.au; 3 ARC Training Centre for Cell and Tissue Engineering Technologies, Queensland University of Technology(QUT), Kelvin Grove, QLD 4059, Australia; 4 Max Planck Queensland Centre for the Materials Science of Extracellular Matrices, Kelvin Grove, QLD 4059, Australia; 5 Centre for Biomedical Technologies, Queensland University of Technology (QUT), 60 Musk Ave., Kelvin Grove, QLD 4059, Australia

**Keywords:** migraine, ion channels, channelopathy, cortical spreading depression, *CHRNA4*, *SLC4A4*, *SLC1A3*, genetics

## Abstract

Dysfunction in ion channels or processes involved in maintaining ionic homeostasis is thought to lower the threshold for cortical spreading depression (CSD), and plays a role in susceptibility to associated neurological disorders, including pathogenesis of a migraine. Rare pathogenic variants in specific ion channels have been implicated in monogenic migraine subtypes. In this study, we further examined the channelopathic nature of a migraine through the analysis of common genetic variants in three selected ion channel or transporter genes: *SLC4A4*, *SLC1A3*, and *CHRNA4*. Using the Agena MassARRAY platform, 28 single-nucleotide polymorphisms (SNPs) across the three candidate genes were genotyped in a case–control cohort comprised of 182 migraine cases and 179 matched controls. Initial results identified significant associations between migraine and rs3776578 (*p* = 0.04) and rs16903247 (*p* = 0.05) genotypes within the *SLC1A3* gene, which encodes the EAAT1 glutamate transporter. These SNPs were subsequently genotyped in an independent cohort of 258 migraine cases and 290 controls using a high-resolution melt assay, and association testing supported the replication of initial findings—rs3776578 (*p* = 0.0041) and rs16903247 (*p* = 0.0127). The polymorphisms are in linkage disequilibrium and localise within a putative intronic enhancer region of *SLC1A3*. The minor alleles of both SNPs show a protective effect on migraine risk, which may be conferred via influencing the expression of *SLC1A3*.

## 1. Introduction

The feedback of neuronal excitability and suppression is essential for maintaining functional brain activities. Elements that affect neuronal management can either lower or elevate the threshold for neuronal firing and may play a role in the pathological cause of a number of neurological conditions including a migraine, epilepsy, stroke, and transient global amnesia [1,2,3,4,5]. An altered threshold may trigger concurrent, self-propagating waves of depolarisation accompanied by massive ion fluxes, which is termed cortical spreading depression (CSD). CSD action originates in the occipital lobe, travels through interconnected neurons and glia, and terminates within the prefrontal cortex [6]. In a normoxic brain, CSD action causes momentary neural suppression, providing an in-built protective mechanism based on negative feedback loops altering excitatory amino acid release, cerebral vascular function and blood flow, cellular metabolism, O_2_ consumption, and protein synthesis [7,8]. In approximately one-third of people with migraines, an aura phase, which may manifest visual or other sensory disturbances, precedes the migraine headache, and is thought to be correlated with CSD [9].

Genetic variants affecting protein function and gene transcription can disrupt the regulation of these processes, and a causative association between a migraine and dysfunction in ion channels and the processes involved in maintaining ionic homeostasis has been found in some rare migraine subtypes [10,11]. In particular, *CACNA1A, ATP1A2*, and *SCN1A* are known causative genes of familial hemiplegic migraine (FHM), a rare autosomal dominant form of a migraine, which features motor aura [12,13,14]. Pathogenic variants in these genes are believed to influence the initiation and propagation of CSD action through hyperexcitability of the neuronal network [15,16,17]: calcium channel variants (*CACNA1A*) have been found to cause a gain-of-function effect in FHM type 1 (FHM1) through enhanced glutamate release and facilitated CSD action [15]; variants in the sodium potassium ATPase gene (*ATP1A2*) conversely cause a loss-of-function effect in FHM type 2 (FHM2) due to extracellular K^+^ accumulation and CSD formation [15,16,17]; and sodium channel (*SCN1A*) variants have been linked to FHM type 3 (FHM3) due to increased firing and hyperexcitability of GABAergic neurons, thereby elevating extracellular K^+^ and prompting CSD initiation [18,19].

In addition to the FHM genes, other genes with roles in neuronal excitability have been associated with familial migraine-related disorders [20,21]. For example, pathogenic variants in *PRRT2*, which are commonly involved in movement disorders, have also been found in hemiplegic migraine families and cases [22]. Similarly, pathogenic variants in the *SLC4A4* and *SLC1A3* genes, encoding astrocytic transporters for bicarbonate and glutamate, respectively, have also been identified in patients with hemiplegic migraines as well as other disorders [23,24,25].

A migraine is a complex neurological disorder with both rare monogenic and more common polygenic forms [21]. With respect to the latter, over 180 susceptibility variants for a migraine have now been identified, largely via genome-wide association studies (GWASs) with increasingly larger and diverse population samples [26,27]. The genes that contribute to polygenic migraine aetiology include those with neural and vascular functions. While the pathogenic variants that cause the rare Mendelian migraine disorders are mainly in genes with ion channel function or involved in ion transport in the brain, recently, some crossover has been observed of the monogenic genes with genes implicated in common migraine susceptibility, e.g., *CACNA1A* and *PRRT2* [27,28]. Given this precedence, it is possible that variants in other ion channel or transporter genes may play a role in susceptibility to common migraine forms. *SLC4A4*, *SLC1A3*, and *CHRNA4* were selected as potential candidate genes for the investigation of polymorphisms in migraine susceptibility.

The *SLC4A4* gene on chromosome (chr) 4q13.3 encodes for the universally expressed sodium/bicarbonate co-transporter, NBCe1. One of its functions is the control of neuronal hyperexcitability by regulating localised synaptic pH levels within connected astrocyte structures [23]. Interestingly, a study by Suzuki et al. examining the genetic basis of proximal renal tubular acidosis (pRTA) identified five rare homozygous variants in *SLC4A4* in patients with pRTA who also had hemiplegic migraines [23]. Functional studies found that constructs carrying these variants in C6 cells revealed a significant correlation between the apparent lack of NBCe1 membrane expression and migraine occurrence [23]. These findings suggest that the near-complete loss of NBCe1 function in astrocytes may cause a migraine through the dysregulation of the synaptic pH, triggering the excessive release and stimulation via neurotransmitters—ultimately leading to neuronal hyperexcitability.

The *SLC1A3* gene (chr 5p13.3) encodes the glutamate transporter Excitatory Amino Acid Transporter 1 (EAAT1). EAAT1 is a master ion channel with two major functions: (i) the regulation of neurotransmitter concentrations at the excitatory glutamatergic synapses within the central nervous system [29]; and (ii) a glutamate-activated anion channel, which directly influences the excitability of presynaptic terminals in certain neurons [30]. An investigation into a patient with a sporadic hemiplegic migraine, seizures, and ataxia identified a variant (P290R) in the *SLC1A3* gene causing neuronal hyperexcitability [24]. Functional expression studies of the P290R variant demonstrated a significant decrease in the expression of the mutated EAAT1 with a markedly reduced capacity for glutamate uptake [24]. Additional in vitro electrophysiological studies illustrated that this variant caused a gain-of-function effect when noticeably increased anion currents in both the presence and absence of glutamate were observed, supporting findings of EAAT1 malfunction in epilepsy [24].

The ion channel gene *CHRNA4* (chr 20q13.33) encodes for an α subunit of the pentameric neuronal nicotinic acetylcholine receptor (nAchR), which is responsible for the control of sodium, potassium, and calcium concentration gradients, membrane depolarisation, and norepinephrine, serotonin, and dopamine release [31,32]. nAchR dysfunction may lower the threshold for CSD initiation and cause central sensitisation through the repeated activation of second- and third-order neurons [33]. When central sensitisation occurs, it is believed to attribute symptoms of allodynia, phonophobia, osmophobia, nausea, and vomiting [34,35,36]. While *CHRNA4* dysfunction has not been previously implicated in a migraine, other studies have attributed its dysfunction to diseases including cognitive dysfunction and attention disorders, epilepsy, depression, Attention Deficit Hyperactivity Disorder (ADHD), Tourette’s syndrome, and other neurodegenerative disorders such as Alzheimer’s disease [37,38]. Interestingly, a study investigating whether the second-generation anticholinesterase drugs may have prophylactic activity in pain disorders found that the acetylcholinesterase inhibitor Donepezil was effective in preventing a migraine [39].

Given the ability of *SLC4A4*, *SLC1A3*, and *CHRNA4* to affect neurotransmission and initiate the progression of neuronal hyperexcitability, we investigated whether common polymorphisms in these three ion channel genes may be associated with a migraine. In this study, a panel of 28 target SNPs from *SLC4A4*, *SLC1A3*, and *CHRNA4* were genotyped in migraine cases and controls and tested for association with migraine susceptibility. The minor alleles of two SNPs within a putative intronic enhancer region of *SLC1A3* were found to show a protective effect on migraine risk.

## 2. Materials and Methods

### 2.1. Sample Populations

Migraine-affected and -unaffected individuals were recruited by the Genomics Research Centre (GRC) from the South East Queensland region through standard advertisement—radio, newspapers, and flyers. All collected samples were of Caucasian origin and diagnosed, by a neurologist, with migraine with aura disorder (MA), with migraine without aura disorder (MO), or as unaffected according to the International Headache Society (IHS) standards of Classification. All migraine samples were matched with a corresponding age-, sex-, and ethnically matched control. Blood collection followed informed participation consent. DNA was extracted and purified from blood using a standard salting-out procedure. From this database, a total of 182 cases and 179 controls were used to complete the Agena MassARRAY genotyping study. A second cohort consisting of 258 cases and 290 controls was used to replicate significant findings. This research was approved by the QUT ethics committee (Ethics Approval Number 1800000611).

### 2.2. Candidate Gene SNP Selection and Genotyping

A total of 28 SNPs across the three genes of interest (*SLC4A4*, *SLC1A3*, *CHRNA4*) were selected based on four principal considerations: (i) literature reports regarding genes/polymorphisms associated with neurological disease, (ii) tagSNPs within the three candidate genes on HapMap [35] with minor allele frequencies below 25%, (iii) functional SNPs predicted by SNPredict2, and (iv) genome-wide associated SNPs highlighted by previous studies. For example, *SLC4A4* rs1041453 was associated in a GWAS with vitamin D-binding protein levels [40] and rs16846575 with vitamin D levels [41]. A multiplex assay was designed using the Assay Design Suite (Agena Bioscience TM). The final design encompassed 12 SNPs in *SLC4A4* (rs2602070, rs2602072, rs4353873, rs4458426, rs10938142, rs1031452, rs1453450, rs12504851, rs16846575, rs4254735, rs9997127, rs1031453), 11 SNPs in *SLC1A3* (rs7728680, rs7729389, rs3776565, rs3776567, rs1428968, rs3776578, rs3776581, rs16903247, rs10491374, rs2269272, rs1529461), and 5 SNPs in *CHRNA4* (rs4809538, rs4522666, rs2273504, rs6010918, rs6122429). Amplification and extension primers were purchased from Integrated DNA Technologies (IDT, Singapore). Primer sequences are available on request. PCR amplification and MassARRAY^®^ genotyping was performed according to the manufacturer’s instructions (Agena Bioscience, San Diego, CA, USA. Briefly, initial genotyping was performed using MassARRAY^®^ matrix-assisted laser desorption ionization time-of-flight (MALDI-TOF) mass spectrometry on a 96-well platform (Agena Bioscience, San Diego, CA, USA). The replication genotyping study was performed using High-Resolution Melt (HRM) protocols with the pre-designed MassARRAY primers according to the manufacturer’s instructions on the QuantStudio 7 Flex Real-Time PCR System (ThermoFisher Scientific, Waltham, MA, USA).

### 2.3. Statistical Analysis

SPSS Statistics 18 (SPSS Inc., Chicago, IL, USA) was used to determine baseline population characteristics. Mean ± SD metrics were determined for the parametric variable age; size and respective population percentages were calculated for the migraine and control cohorts in terms of gender and migraine subtypes. PLINK v1.7 [36] was used for the statistical analysis of all genotypic data. Two SNPs (rs1031453 and rs3776581) were removed from a further analysis due to strong deviations from Hardy–Weinberg Equilibrium [HWE > 0.05]. Pearson’s chi-square tests were used to determine significant associations between selected SNPs and a migraine. A logistic regression analysis was performed to minimise covariate (age/sex) influence. LDlink [37] was then used to calculate linkage disequilibrium (LD) associations between gene-specific SNPs.

## 3. Results

### 3.1. Characteristics of Study Cohorts

The demographics for the initial (cohort 1, N = 361) and replication (cohort 2, N = 548) case–control cohort can be viewed in Table 1. In cohort 1, mean age was 44 and females were predominant in both the migraine population (N = 144, 79.1%) and control population (N = 121, 67.6%). In cohort 2, both cases and controls involved a prominent number of females (~86%) and a mean age of 54. The distribution of gender across the two populations closely resembles the literature-defined 3 females/1 male migraine prevalence ratio [38].

### 3.2. Genotyping of 26 SNPs across SLC4A4, SLC1A3, and CHRNA4 in Migraine Samples versus Controls

The Agena MassARRAY genotyping plex was run with cohort 1 DNA samples. Genotype details and frequencies of examined SNPs in *SLC4A4*, *SLC1A3*, and *CHRNA4* are included in Table 2. Proportions of wildtype homozygotes (AA), heterozygotes (AB), and mutant homozygotes (BB) were compared within migraine and control samples for each SNP.

Results from Pearson’s chi-square test of association for the SNP alleles tested are summarized in Table 3. Two SNPs, rs3776578 [X^2^ (1, N = 361) = 4.54, *p* = 0.03311] and rs16903247 [X^2^ (1, N = 361) = 3.891, *p* = 0.04855], located in gene *SLC1A3* on chromosome 5, were found to show a significant association (α ≤ 0.05) with unaffected individuals when compared to those with a migraine. The odds ratios (ORs) for the two SNPs rs3776578 and rs16903247 are 0.4285 (95% CI = 0.192, 0.954) and 0.4523 (95% CI = 0.201, 1.014), respectively. An OR < 1 indicates that the minor allele ‘C’ reduces disease risk and incurs a protective function in both instances.

Both the rs3776578 and rs16903247 SNPs were found to be in Hardy–Weinberg Equilibrium (*p* > 0.05) and the minor allele frequency was significantly higher in healthy individuals (Pearson’s chi-square test of association ≤ 0.05). To avoid age effects and gender bias when assessing the sole protective effect of each polymorphism, a logistic regression analysis was performed using age and gender as covariates, summarized in Table 4. The rs3776578 (*p*-value = 0.0338) and rs16903247 (*p* = 0.049) SNPs were found to be significantly associated with healthy individuals (α ≤ 0.05) under the unadjusted model. However, when adjusted for effects of age and gender, rs16903247 was no longer significant (*p* = 0.056), while rs3776578 remained significantly associated (*p* = 0.043) with healthy individuals. Interestingly, a significant association was identified for both SNPs with sex and a migraine [rs16903247 *p* = 0.014, rs3776578 *p* = 0.016], with this correlation previously reported [42]. Smoking has also been reported to be causally associated with risk of developing a migraine [43]; however, this information was not available for participants to be able to add it as a covariate in this analysis.

### 3.3. Linkage Disequilibrium Analysis

The linkage disequilibrium (LD) analysis was conducted to determine LD between target SNPs rs3776578 and rs16903247 and between target SNPs and other rare SNPs within *SLC1A3* (MAF < 0.05). There were strong LD associations—a D’ value of 1.0 and an r^2^ correlation value of 0.960 were identified between the rs3776578 and rs16903247 SNPs. The examination of the LD association analysis between rs3776578 and rs16903247 and other rare SNPs within *SLC1A3* identified mild-to-moderate LD associations (D’ > 0.80, r^2^ > 0.40) with five additional *SLC1A3* intronic SNPs (MAF > 0.05): rs3776563, rs3836803, rs79029484, rs3756468, and rs3776583.

### 3.4. Replication Analysis

Genotyping for significant SNPs rs3776578 and rs16903247 was undertaken in a larger, independent migraine case–control cohort using an HRM assay. For SNP rs3776578, 252 cases and 267 controls had the wildtype ‘TT’ and 6 cases and 23 controls had the heterozygous ‘TC’ genotype; no mutant ‘CC’ genotypes were detected in either cases or controls. For SNP rs16903247, 252 cases and 270 controls had the wildtype ‘TT’ and 6 cases and 20 controls had the heterozygous ‘TC’ genotype, with no mutant ‘CC’ genotypes found in either cases or controls. Pearson’s chi-square test on the replication cohort is summarised in Table 5. The *SLC1A3* SNPs rs3776578 [X^2^ (1, N = 548) = 8.232, *p* = 0.0041] and rs16903247 [X^2^ (1, N = 548) = 6.197, *p* = 0.0127] were found to be significantly associated with migraine disorder at α ≤ 0.05. Furthermore, the odds ratio for both SNPs was <1, which supports the finding from the initial study cohort that the minor ‘C’ allele is protective with respect to migraine risk.

A haplotype analysis was then used to further confirm rs3776578 and rs16903247 migraine association. Genotyping data from cohort 1 and 2 were combined for this analysis (N = 909). As seen in Table 6, both haplotypes H1 (CC) and H2 (TT) formed by SNPs rs3776578 and rs16903247 [X^2^ (1, N = 909) = 8.866, *p* = 0.002] were significantly associated with migraine (α ≤ 0.01), supporting the results observed for the SNP associations.

## 4. Discussion

The examination of common SNPs within candidate genes *SLC4A4*, *SLC1A3*, and *CHRNA4*, and their relationship to migraine susceptibility, found significant associations for two *SLC1A3* gene SNPs, rs3776578 and rs16903247. These two SNPs were found to be in high LD, and odds ratios for each suggested a protective effect of the minor allele variant with respect to migraine susceptibility. No statistically significant associations were detected for the other two candidate ion channel genes *CHRNA4* and *SLC4A4.* Additional genotyping of *SLC1A3* rs16903247 and rs3776578 variants in a replication case–control cohort further supported association between the minor alleles at the SNP and reduced migraine risk.

The *SLC1A3* gene encodes a Na^+^/K^+^-dependent glutamate transporter, which plays an integral role in excitatory neurotransmission regulation and downstream antioxidant synthesis, and actively provides protection against localised excitotoxic damage [44]. Functionally, the EAAT1 receptor regulates neuronal glutamate-dependent excitation through secondary-active glutamate transport to recapture glutamate from the synaptic cleft, while also simultaneously facilitating ion regulation as a Na^+^/K^+^ ion channel [45]. Rare pathogenic genetic variations within *SLC1A3* have been found to be causal for episodic ataxia, type 6 (OMIM #612656), and may be associated with other central nervous system (CNS) pathologies, including epilepsy and a hemiplegic migraine [24,25,46,47]. Our findings suggest that common SNPs in *SLC1A3* may also play a role in susceptibility to the more common migraine type with a polygenic basis.

Although the functional effects of both implicated intronic SNP sites are unknown, in silico prediction methods may suggest a mechanism behind the association. Imputation of the two SNP sites into Ensembl’s Variant Effect predictor platform [48] revealed that both intronic SNPs rs3776578 and rs16903247 are predicted to have a ‘modifier impact’ within an enhancer regulatory region. This is further supported by the observed histone H3 lysine 27 acetylation marks and clusters of DNaseI hypersensitivity documented in the ENCODE database [49]. According to the University of California Santa Cruz (UCSC) genome browser CAVIAR track [50], which presents high-confidence sets of genetic variants that are expression quantitative trait loci (eQTLs), SNPs in the region encompassing rs3776578 and rs16903247 affect *SLC1A3* expression in a range of tissues. Furthermore, the JASPAR CORE 2024 track shows that rs16903247 is located in predicted transcription factor binding sites for ZNF214 and HNF1A, with the SNP site directly affecting the consensus binding site for the latter. It is therefore possible that these SNPs affect migraine susceptibility via influencing either alternative mRNA splicing or expression levels of *SLC1A3* transcripts.

The finding of strong LD between both SNPs supports not only the integrity of the genotyping results but also supports the premise that protective SNPs within *SLC1A3* gene enhancer regions play a role in limiting migraine susceptibility. Correlations between polymorphisms in enhancer regions and effects on gene expression have been noted in numerous functional studies. For example, an interesting study of the human renin gene found that two SNPs in strong LD, located within an enhancer region, were associated with a 45% increase in enhancer activity [51].

A study conducted by de Vries et al. (2016) evaluated genes from candidate gene association studies in migraines using the International Headache Genetics Consortium (IHGC) genome-wide association (GWA) data set (5175 patients with a migraine and 13,972 controls) [52]. The study focused on 21 genes from published candidate gene association studies and 6 additional genes from other non-GWA studies in migraines, which included the *SLC1A3* gene. Results from this study found no SNPs in or near the 27 targeted genes, including SNPs previously identified as migraine-associated, to be statistically significant after Bonferroni correction [52]. De Vries et al. suggests that the lack of the replication of genetic markers may be a result of a low power and small effect size in candidate gene association studies, or that the GWAS was unable to sufficiently reflect specific LD patterns [52], either of which may apply to our targeted SNPs of interest, rs3776578 and rs16903247.

Despite the lack of supporting association evidence of rs3776578 and rs16903247 in migraine GWASs, SNP correlations with biological processes can be drawn from a GWAS performed on the British 1958 birth cohort [15]. Genotyping data were subjected to a quantitative trait association analysis based on measurements collected during a follow-up study by Strachan et al. in 2007 [53]. The quantitative trait analysis identified 959,443 significant markers in the GWAS central database, including nominal associations between SNPs rs3776578 (*p* = 0.0004) and rs16903247 (*p* = 0.001) and blood plasma fibrinogen levels. Therefore, the same *SLC1A3* SNPs we identified as associated with a migraine are also associated with blood fibrinogen levels. Interestingly, relationships between fibrinogen levels and migraine susceptibility have been reported previously, although not always consistent; nevertheless, a two-sample Mendelian randomization study found a causal relationship between genetically predicted decreased fibrinogen and a migraine, particularly a migraine with aura [54].

A limitation of this study was that only a limited number of variants and ion channel or ion transporter-related genes were considered. While SNPs in the *CHRNA4* locus were investigated in the current study, *CHRNA7* may be a better target as some recent findings suggest that α7nAChR channels may play a role in a migraine, modulating pain and inflammatory pathways via the attenuation of glial and astrocytic cell activation [55,56].

## 5. Conclusions

In summary, we have identified associations between *SLC1A3* polymorphisms (rs3776578 and rs16903247) and migraine risk. When we consider the location of both polymorphisms and the neuroprotective function of the *SLC1A3* gene, it is conceivable that an enhanced gene function due to genotypic variation may increase the threshold for CSD action, thereby affecting and reducing susceptibility to migraine attacks. Our results suggest that further studies examining additional *SLC1A3* SNPs, or other neuroprotective ion channel genes, may provide insight into migraine aetiology.

## Figures and Tables

**Table 1:** Descriptive characteristics of cohorts 1 and 2. Cohort 1 was used in the initial MassARRAY genotyping study and cohort 2 was used in the replication genotyping study.

**Cohort 1**		
	Migraine N = 182	Control N = 179
Female N, (%)	144 (79.1)	121 (67.6)
Male N, (%)	38 (20.9)	58 (32.4)
Age (years), mean ± SD	44 ± 16	44 ± 14
Migraine with aura	104 (57.1)	-
Migraine without aura	78 (42.9)	-
**Cohort 2**		
	Migraine N = 258	Control N = 290
Female N, (%)	221 (85.6)	250 (86.2)
Male N, (%)	37 (14.4)	40 (13.8)
Age (years), mean ± SD	54 ± 13	54 ± 14
Migraine with aura	213 (82.5)	-
Migraine without aura	45 (17.5)	-

N—Number, SD—Standard Deviation.

**Table 2:** Prevalence of 26 SNPs across *SLC4A4*, *SLC1A3*, and *CHRNA4* in migraine versus controls.

SNP	Chromosome Position *	Allele		Wildtype Homozygotes, AA (%)		Heterozygotes, AB (%)		Mutant Homozygotes, BB (%)	
		A	B	Migraine	Control	Migraine	Controls	Migraine	Controls
*SLC4A4*	Chr 4:								
rs2602070	71,250,885	C	A	180 (98.9)	173 (96.6)	2 (1.1)	6 (3.4)	0 (0.0)	0 (0.0)
rs2602072	71,256,316	G	T	179 (98.4)	172 (96.1)	3 (1.6)	7 (3.9)	0 (0.0)	0 (0.0)
rs4353873	71,428,816	A	G	123 (67.6)	123 (69.5)	54 (29.7)	49 (27.7)	5 (2.7)	5 (2.8)
rs4458426	71,475,673	T	A	116 (63.7)	124 (69.3)	59 (32.4)	51 (28.5)	7 (3.8)	4 (2.2)
rs10938142	71,496,462	G	A	154 (84.6)	147 (82.1)	28 (15.4)	29 (16.2)	0 (0.0)	3 (1.7)
rs1031452	71,519,326	C	T	95 (52.2)	90 (50.6)	74 (40.7)	78 (43.8)	13 (7.1)	10 (5.6)
rs1453450	71,533,996	C	A	140 (76.9)	133 (74.3)	38 (20.9)	41 (22.9)	4 (2.2)	5 (2.8)
rs12504851	71,545,167	A	G	112 (61.5)	103 (57.9)	60 (33.0)	61 (34.3)	10 (5.5)	14 (7.9)
rs16846575	71,555,992	T	C	118 (64.8)	116 (64.8)	56 (30.8)	60 (33.5)	8 (4.4)	3 (7.7)
rs4254735	71,564,768	T	C	179 (98.9)	175 (97.8)	2 (1.1)	4 (2.2)	0 (0.0)	0 (0.0)
rs9997127	71,570,781	A	G	180 (98.9)	175 (97.8)	2 (1.1)	4 (2.2)	0 (0.0)	0 (0.0)
*SLC1A3*	Chr 5:								
rs7728680	36,612,951	A	G	88 (48.4)	80 (44.7)	78 (42.8)	72 (40.2)	16 (8.8)	27 (15.1)
rs7729389	36,613,099	A	G	117 (64.3)	116 (64.8)	58 (31.9)	57 (31.8)	7 (3.8)	6 (3.4)
rs3776565	36,631,551	T	C	123 (67.6)	129 (72.1)	54 (29.7)	44 (24.6)	5 (2.7)	6 (3.3)
rs3776567	36,632,373	A	C	124 (84.3)	137 (91.3)	22 (15.0)	13 (8.7)	1 (0.7)	0 (0.0)
rs1428968	36,646,844	C	T	133 (73.5)	127 (71.3)	45 (24.9)	43 (24.2)	3 (1.6)	8 (4.5)
rs3776578	36,651,884	T	C	173 (95.1)	159 (88.8)	9 (4.9)	20 (11.2)	0 (0.0)	0 (0.0)
rs16903247	36,653,353	T	C	173 (95.1)	160 (89.4)	9 (4.9)	19 (10.6)	0 (0.0)	0 (0.0)
rs10491374	36,667,477	C	G	48 (26.4)	45 (25.3)	93 (51.1)	90 (50.6)	41 (22.5)	43 (24.2)
rs2269272	36,687,754	C	T	112 (61.9)	120 (67.0)	65 (35.9)	49 (27.4)	4 (2.2)	10 (5.6)
rs1529461	36,689,261	G	A	104 (57.1)	116 (64.8)	72 (39.6)	55 (30.7)	6 (3.3)	8 (4.5)
*CHRNA4*	Chr 20:								
rs4809538	63,338,824	G	A	102 (56.4)	100 (56.8)	73 (40.3)	69 (39.2)	6 (3.3)	7 (4.0)
rs4522666	63,343,128	A	G	71 (39.0)	64 (35.7)	93 (51.1)	90 (50.3)	18 (9.9)	25 (14.0)
rs2273504	63,356,709	G	A	128 (70.3)	120 (67.4)	52 (28.6)	52 (29.2)	2 (1.1)	6 (3.4)
rs6010918	63,358,149	G	A	116 (95.1)	103 (88.8)	6 (4.9)	13 (11.2)	0 (0.0)	0 (0.0)
rs6122429	63,361,854	C	T	134 (73.6)	133 (74.3)	45 (24.7)	44 (24.6)	3 (1.6)	2 (1.1)

SNP—Single-nucleotide polymorphism, *SLC4A4*—Solute carrier family 4 member 4, *SLC1A3*—Solute carrier family 1 member 3, *CHRNA4*—Cholinergic receptor nicotinic α 4 subunit, N—Number, A—Major allele, B—Minor allele; * genomic positions refer to GRCh38.p7/hg38 assembly.

**Table 3:** Association testing of *SLC4A4*, *SLC1A3,* and *CHRNA4* SNPs with migraine using Pearson’s Chi-Square tests.

SNP	Allele		F_A	F_U	X^2^	*p*-Value	OR	L95	U95
	A	B							
*SLC4A4*									
rs2602070	C	A	0.0054	0.0167	2.0900	0.1482	0.3241	0.064	1.617
rs2602072	G	T	0.0082	0.0195	1.6910	0.1935	0.4167	0.106	1.624
rs4353873	A	G	0.1758	0.1667	0.1060	0.7447	1.0670	0.723	1.573
rs4458426	T	A	0.2005	0.1648	1.5440	0.2141	1.2710	0.870	1.857
rs10938142	G	A	0.0769	0.0977	0.9844	0.3211	0.7690	0.457	1.293
rs1031452	C	T	0.2747	0.2753	0.0002	0.9867	0.9972	0.718	1.383
rs1453450	C	A	0.1264	0.1425	0.4015	0.5263	0.8708	0.567	1.336
rs12504851	A	G	0.2198	0.2500	0.9150	0.3388	0.8451	0.598	1.193
rs16846575	T	C	0.1978	0.1844	0.2110	0.6460	1.0910	0.752	1.581
rs4254735	T	C	0.0055	0.0111	0.6949	0.4045	0.4917	0.089	2.701
rs9997127	A	G	0.0054	0.0111	0.7062	0.4007	0.4890	0.088	2.686
*SLC1A3*									
rs7728680	A	G	0.3022	0.3520	2.0310	0.1541	0.7974	0.583	1.089
rs7729389	A	G	0.1978	0.1927	0.0294	0.8637	1.0330	0.714	1.492
rs3776565	T	C	0.1758	0.1564	0.4902	0.4839	1.1500	0.776	1.704
rs3776567	A	C	0.0816	0.0433	3.7290	0.0534	1.9620	0.979	3.932
rs1428968	C	T	0.1409	0.1657	0.8541	0.3554	0.8255	0.549	1.24
rs3776578	T	C	0.0247	0.0558	4.5400	0.0331 *	0.4285	0.192	0.954
rs16903247	T	C	0.0247	0.0530	3.8910	0.0485 *	0.4523	0.201	1.014
rs10491374	C	G	0.4808	0.4944	0.1335	0.7148	0.9470	0.684	1.279
rs2269272	C	T	0.2017	0.1927	0.0904	0.7636	1.0580	0.706	1.268
rs1529461	G	A	0.2308	0.1983	1.1270	0.2884	1.2130	0.732	1.527
*CHRNA4*									
rs4809538	G	A	0.2348	0.2358	0.0009	0.9752	0.9945	0.849	1.732
rs4522666	A	G	0.3544	0.3911	1.0380	0.3083	0.8548	0.703	1.405
rs2273504	G	A	0.1538	0.1798	0.8712	0.3506	0.8295	0.632	1.156
rs6010918	G	A	0.0245	0.0537	2.7440	0.0976	0.4441	0.560	1.229
rs6122429	C	T	0.1401	0.1341	0.0555	0.8138	1.0520	0.166	1.188
rs6122429	C	T	0.1401	0.1341	0.0555	0.8138	1.0520	0.688	1.608

SNP—Single-Nucleotide Polymorphism, *SLC4A4*—Solute Carrier Family 4 Member 4, *SLC1A3*—Solute Carrier Family 1 Member 3, *CHRNA4*—Cholinergic Receptor Nicotinic α 4 Subunit, A—Major Allele, B—Minor Allele, F_A—Frequency of Minor Allele in Migraine, F_U—Frequency of Minor Allele in Controls, X^2^—Chi-Square Value, OR—Odds Ratio, L95—Lower Bound of 95% Confidence Interval, U95—Upper Bound of 95% Confidence Interval. * Meets significance threshold (α ≤ 0.05).

**Table 4:** Logistic regression analysis results, adjusting for age/sex and independently determining covariate association with disease.

TEST	OR	SE	L95	U95	STAT	*p*-Value
*SLC1A3* rs16903247						
Logistic Regression (Unadjusted)	0.4381	0.4193	0.1926	0.996	−1.969	0.0490 *
Adjusted by Sex and Age	0.447	0.4229	0.1951	1.024	−1.904	0.0569
Sex Association with Disease	1.812	0.2443	1.1220	2.924	2.433	0.01497 *
Age Association with Disease	1.006	0.0078	0.9908	1.022	0.787	0.4310
*SLC1A3* rs3776578						
Logistic Regression (Unadjusted)	0.4136	0.4161	0.1830	0.935	−2.122	0.03387 *
Adjusted by Sex and Age	0.4282	0.4198	0.1881	0.975	−2.020	0.04336 *
Sex Association with Disease	1.797	0.2445	1.1130	2.902	2.397	0.01654 *
Age Association with Disease	1.006	0.0078	0.9908	1.022	0.778	0.4360

SNP—Single-nucleotide polymorphism, *SLC1A3*—Solute carrier family 1 member 3, OR—Odds ratio, SE—Standard error, L95—Lower bound of 95% confidence interval, U95—Upper bound of 95% confidence interval, STAT—Coefficient *t*-statistic. * Meets significance threshold (α ≤ 0.05).

**Table 5:** Results from Pearson’s chi-square test of association performed on the replication cohort.

SNP	Allele		F_A	F_U	X^2^	*p*-Value	OR	L95	U95
	A	B							
*SLC1A3*									
rs3776578	T	C	0.0119	0.0404	8.232	0.0041 *	0.2866	0.1158	0.7907
rs16903247	T	C	0.0116	0.0347	6.197	0.0127 *	0.3283	0.1308	0.8241

SNP—Single-Nucleotide Polymorphism, *SLC1A3*—Solute Carrier Family 1 Member 3, A—Major Allele, B—Minor Allele, F_A—Frequency of Minor Allele in Migraine, F_U—Frequency of Minor Allele in Controls, X^2^—Chi-Square Value, OR—Odds Ratio, L95—Lower Bound of 95% Confidence Interval, U95—Upper Bound of 95% Confidence Interval. * Meets significance threshold (α ≤ 0.05).

**Table 6:** Results from haplotype analysis performed on the combined genotyping data from cohort 1 and cohort 2.

SNPs	Haplotype	F_A	F_U	X^2^	DF	*p*-Value
rs16903247/rs3776578	H1-CC	0.018	0.042	8.866	1	0.002 *
	H2-TT	0.982	0.958	8.866	1	0.002 *
	H3-TC	0.000	0.000	-	-	-
	H4-CT	0.000	0.000	-	-	-

SNP—Single-Nucleotide Polymorphism, H1—Haplotype 1, H2—Haplotype 2, H3—Haplotype 3, H4—Haplotype 4, F_A—Frequency of Minor Allele in Migraine, F_U—Frequency of Minor Allele in Controls, X^2^—Chi-Square Value, DF—Degree of Freedom. * Meets significance threshold (α ≤ 0.01).

## Data Availability

The original contributions presented in the study are included in the article, further inquiries can be directed to the corresponding author.
